# The CLV-WUS Stem Cell Signaling Pathway: A Roadmap to Crop Yield Optimization

**DOI:** 10.3390/plants7040087

**Published:** 2018-10-19

**Authors:** Jennifer C. Fletcher

**Affiliations:** 1 Plant Gene Expression Center, United States Department of Agriculture-Agricultural Research Service, Albany, CA 94710, USA; jfletcher@berkeley.edu; Tel.: +1-510-559-5917; Fax: +1-510-559-5678; 2 Department of Plant and Microbial Biology, University of California, Berkeley, CA 94720, USA

**Keywords:** CLE, CLV, WUS, stem cells, meristem, SAM, signaling, locule

## Abstract

The shoot apical meristem at the growing shoot tip acts a stem cell reservoir that provides cells to generate the entire above-ground architecture of higher plants. Many agronomic plant yield traits such as tiller number, flower number, fruit number, and kernel row number are therefore defined by the activity of the shoot apical meristem and its derivatives, the floral meristems. Studies in the model plant *Arabidopsis thaliana* demonstrated that a molecular negative feedback loop called the CLAVATA (CLV)-WUSCHEL (WUS) pathway regulates stem cell maintenance in shoot and floral meristems. CLV-WUS pathway components are associated with quantitative trait loci (QTL) for yield traits in crop plants such as oilseed, tomato, rice, and maize, and may have played a role in crop domestication. The conservation of these pathway components across the plant kingdom provides an opportunity to use cutting edge techniques such as genome editing to enhance yield traits in a wide variety of agricultural plant species.

## 1. Introduction

Plants are unique among living organisms in their ability to continuously grow and develop new organs throughout their life cycles. This continuous growth strategy produces leaves, stems, and flowers in architectures that can vary widely between species, from squat yellow dandelions to tall, leafy trees. The sources of cells for continuous organ formation are the apical meristems at the growing shoot and root tips. The shoot apical meristem (SAM) forms in the embryo and consists of a small reservoir of stem cells whose descendants generate all of the above-ground structures of the plant [1]. Following germination, the vegetative SAM produces a series of leaves from its flanks. At the transition to flowering the vegetative meristem becomes a reproductive inflorescence meristem (IFM) that produces axillary meristems followed by floral meristems that generate the flowers and seeds. Thus, SAM activity is the ultimate source of many yield traits in agronomic crop plants, because the direct outcome of plant organogenesis is the production of leaves, fruits, pods, seeds, and other structures that humans harvest and eat.

**Figure 1:**
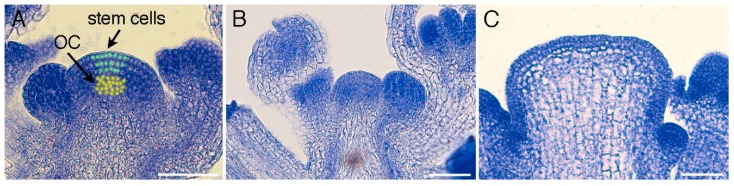
Shoot apical meristems of *Arabidopsis* wild-type and *clv3* mutant plants during the inflorescence phase. (**A**) Key domains within the shoot apical meristem. The apical stem cells are colored in green and the underlying organizing center (OC) cells in yellow. Primordia arise as dome-shaped structures on the meristem flanks. (**B**) Wild-type Columbia-0 inflorescence meristem (IFM) and flanking floral meristem primordia. (**C**) Enlarged *clv3* null mutant IFM and flanking floral meristem primordia. Scale bars, 50 μm.

The SAM has the dual function of maintaining an active stem cell population while concurrently generating new organs. The organs form as primordia on the meristem flanks, while the self-renewing stem cell reservoir at the apex replenishes the cells that depart from the meristem into the primordia (Figure 1A). The stem cell pool is sustained by the activity of an underlying group of cells in the core of the SAM called the organizing center (OC). The maintenance of SAM homeostasis via a balance between stem cell loss and renewal is critical for plant development, because plants with reduced SAM activity prematurely cease growth before forming their full complement of organs [2,3] whereas those with over-active meristems have enlarged stems and can produce many extra branches, flowers, fruits, and seeds [4,5].

Communication between individual cells is crucial to coordinate the various aspects of SAM function. Classical experiments demonstrated that the fate of each SAM cell is determined by positional information rather than by its lineage-specific heritage [6,7,8], and that the distinct functional domains within the SAM exchange cell fate information cues [9]. The SAM is further stratified into clonally distinct cell layers [10,11,12] that participate in both SAM maintenance and organ formation [13,14], requiring that these activities be orchestrated between all cell layers. Therefore, signaling between SAM cells is necessary for the cells to assess their relative positions in the meristem and behave coordinately with their neighbors. As described below, a molecular network called the CLAVATA (CLV)-WUSCHEL (WUS) pathway conveys intercellular signals that are critical for shoot and floral meristem maintenance in higher plants.

Crop plants have undergone vigorous selection by humans during the past 10,000 years [15,16], especially for yield traits such as larger and more numerous inflorescence meristems, fruits, and seeds. The CLV-WUS pathway in particular has been a target of selection during crop domestication to enhance agricultural yields [17]. Here, I review our understanding of the CLV-WUS signaling system in *Arabidopsis* shoot meristems and discuss studies demonstrating that components of the pathway are associated with variation in yield traits in agronomic crops such as mustard, tomato, rice, and maize.

## 2. CLV-WUS Shoot Apical Meristem Maintenance Pathway

**Figure 2:**
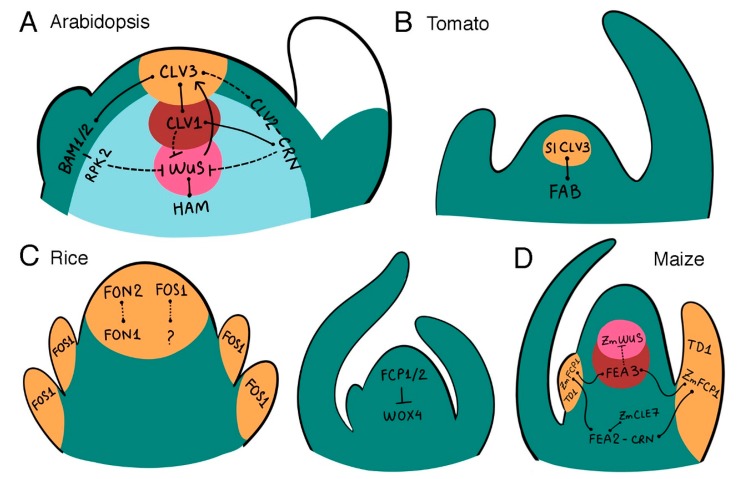
CLV-WUS signaling pathways in model and crop plant meristems. (**A**) *Arabidopsis* SAM. (**B**) Tomato SAM. (**C**) Rice FM and SAM. (**D**) Maize SAM. Genes with characterized genetic and/or biochemical interactions are shown. Arrows depict positive regulation and bars depict negative regulation. Solid lines represent direct interactions and dashed lines represent indirect interactions. Solid lines with rounded ends depict direct peptide–receptor interactions. Unidentified receptors for peptides are denoted by question marks.

The CLV-WUS signaling pathway plays a central role in maintaining shoot and floral stem cell homeostasis in *Arabidopsis* (Figure 2A). The *WUS* gene is dispensable for establishing the embryo stem cell reservoir [18], but is required to sustain stem cell fate during vegetative and reproductive development [3]. *WUS* is expressed exclusively in the SAM organizing center and encodes a homeodomain transcription factor of the WUSCHEL-LIKE HOMEOBOX (WOX) family [19]. WUS is a bi-functional protein that can both repress and activate gene transcription in the SAM [20]. Among the key targets of direct WUS repression in the OC are negative regulators of cytokinin activity, a hormone that promotes cell proliferation across the SAM [21]. WUS also directly represses the transcription of cell differentiation-inducing transcription factor genes that are normally expressed in organ primordia, to prevent premature stem cell differentiation at the apex of the SAM [22]. In addition, WUS protein moves between cells through plasmodesmata into the apical stem cell domain [23] where it maintains stem cell fate and induces the expression of the *CLV3* gene in a dosage-dependent fashion [24,25]. WUS functions together with members of the HAIRY MERISTEM (HAM) family of GRAS domain transcriptional regulators to regulate stem cell production [26] and to ensure that *CLV3* transcription is activated exclusively in the outermost apical layers of the SAM [27].

The CLV signal transduction pathway negatively regulates stem cell accumulation in above-ground meristems. Mutations in *Arabidopsis CLV* genes cause progressive enlargement of the shoot and floral stem cell pools (Figure 1B,C), resulting in plants with enlarged stems and excess flowers, as well as flowers with extra sepals, petals and stamens, and siliques with more than two locules [4,28]. *CLV3* encodes a founding member of the CLAVATA3/EMBRYO SURROUNDING REGION (CLE) family of polypeptides [29], which are present throughout the plant kingdom [30,31]. *CLV3* is expressed within the shoot and floral stem cell domain [32] and encodes a pre-propeptide that is processed into a 12–13 amino acid arabinosylated glycoprotein [33,34]. This glycoprotein moves through the extracellular space to communicate stem cell fate information with neighboring cells [35].

**Figure 3:**
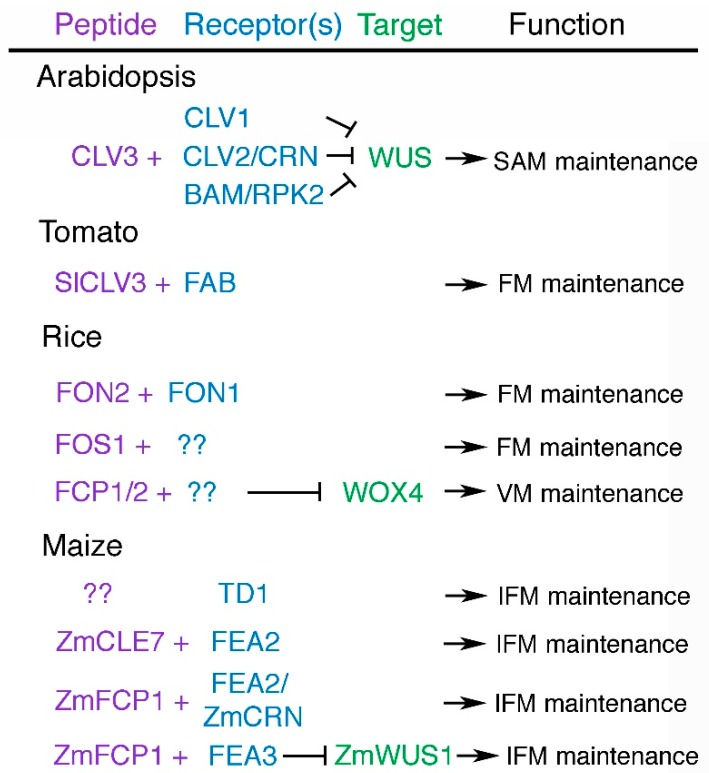
Components of CLV-WUS signaling pathways and their functions in model and crop plants. Proteins with characterized genetic and/or biochemical interactions are listed. Unidentified peptides and receptors are denoted by question marks. Arrows depict positive regulation and bars depict negative regulation. SAM, shoot apical meristem; FM, floral meristem; VM, vegetative meristem; IFM, inflorescence meristem.

The CLV3 signal is perceived and transduced at the plasma membrane by several distinct sets of receptors (Figure 2A and Figure 3). CLV3 peptides are bound by the CLV1 leucine-rich repeat receptor-like kinase (LRR-RLK) that is produced in cells beneath the stem cell reservoir [36,37]. A second distinct receptor complex consists of heterodimers of the CLV2 LRR receptor-like protein [38] and the CORYNE (CRN) protein, a presumptive pseudokinase that functions as a CLV2 co-receptor [39,40]. CRN mediates localization of CLV2/CRN complexes to the plasma membrane [41], where they can directly interact with CLV1 heterodimers [41,42,43]. Yet in contrast to *CLV1*, *CLV2* and *CRN* are expressed throughout the entire SAM, and the CLV2-CRN complex functions largely independently of CLV1 in CLV3 signal transduction [39,41,43]. Reports differ as to whether the CLV2 receptor itself directly binds the CLV3 ligand or if an additional co-receptor is required [42,44]. Other receptors appear to mediate CLV3 signaling predominantly on the flanks of the meristem. Three LRR-RLK genes that form a monophyletic group with *CLV1*, termed *BARELY ANY MERISTEM1*, *2* and *3 (BAM1–3)*, act redundantly to promote stem cell maintenance on the meristem periphery [45], and both BAM1 and BAM2 directly bind CLV3 peptides [42,44]. The BAM1 protein physically associates with the LRR receptor-like kinase RECEPTOR-LIKE PROTEIN KINASE2 (RPK2) [46], which itself does not bind CLV3 peptides and thus is proposed to regulate meristem maintenance by transmitting the CLV3 signal through the BAM1 pathway [44]. An additional group of four LRR-RLKs termed the CLAVATA3 INSENSITIVE RECEPTOR KINASES (CIKs) undergo rapid phosphorylation in response to CLV3 signaling, and appear to function as co-receptors for the CLV1, CLV2-CRN, and BAM-RPK2 receptor pathways [47]. CLV3-mediated signaling through these receptor complexes limits stem cell accumulation by restricting the *WUS* expression domain to the OC [48,49]. Thus, the CLV-WUS pathway functions as a dynamic negative feedback loop that allows the stem cell domain and the underlying OC to continually adjust their size relative to one another to maintain SAM homeostasis.

## 3. CLV-WUS Pathway in Dicotyledonous Crop Plants

*Arabidopsis thaliana* is related to cultivated mustard varieties—such as *Brassica rapa*, *Brassica juncea*, and *Brassica napus*—which are agriculturally important oil crops that provide edible oils for human diets as well as raw material for animal feed and industrial processes such as biodiesel production [50]. Like *Arabidopsis*, oilseed floral meristems produce sepals, petals, stamens, and two carpels, the latter of which develop into the two locules of the siliques. The oil and protein products of Brassica plants are contained inside the seeds that develop within the siliques, and thus enhancing silique yield traits has long been a major goal of oilseed production and genetic improvement [51,52].

Several multilocular Brassica lines with more than two locules have been identified in natural populations [53], and recent studies have implicated CLV-WUS pathway components in the appearance of this trait (Table 1). The *B. rapa* var. *yellow sarson ml4* mutant exhibits a multilocular phenotype caused by a single nucleotide mutation in a *CLV3* gene homolog that produces an amino acid substitution in the CLE domain [54]. Similarly, a multilocular phenotype found in the *B. juncea* Duoshi cultivar results from mutations in a *CLV1* gene homolog, *BjLn1* [55,56], while a trilocular phenotype in *B. juncea* J163-4 plants is caused by the insertion of a copia-LTR retrotransposable element into the coding region of a second *CLV1* homolog, *BjMc1*, interrupting its transcription [52]. These multilocular Brassica plants have significantly higher yield than the corresponding bilocular plants without affecting viability [54,56,57], suggesting that selectively targeting *CLV* genes can be a powerful method of obtaining high-yield oilseed cultivars. This has been tested by the use of CRISPR-Cas9 genome editing to target *CLV* pathway components in allotetraploid *B. napus* plants, which contain two copies each of the *CLV1*, *CLV2*, and *CLV3* genes [50]. Simultaneous mutation of both copies of any of the three *BnCLV* genes resulted in plants with enlarged IFMs, multilocular siliques, and higher seed yield, with mutations in the *BnCLV3* genes producing the most severe effects [50].

Members of the CLV-WUS pathway also play key roles in regulating locule number in tomato (Table 1, Figure 2B). The wild ancestor of tomato had a small, bilocular fruit, whereas modern tomato varieties contain eight or more locules [64]. The *fasciated (fas)* and *locule number (lc)* genes are the major quantitative trait loci (QTL) controlling the number of tomato fruit locules, and most cultivated tomato varieties contain mutations in either the *fas* or the *fas* and *lc* genes [65]. The multilocular *fas* phenotype results from a mutation in the regulatory region of a *CLV3*-related gene, *SlCLV3* [59], whereas the *lc* trait is caused by two single nucleotide polymorphisms (SNPs) in a repressor element downstream of a *WUS* gene homolog [66,67]. Evidence suggests that selection at both loci took place during tomato domestication to produce plants with increased fruit locule number [59,66]. Generation of a suite of novel *SlCLV3* promoter alleles using genome editing produced plants with a continuum of variation in fruit locule number [67], providing a blueprint for engineering quantitative variation in yield traits for breeding purposes.

In addition, a forward genetic screen for tomato mutants with increased inflorescence branching and fruit locule number identified both known and novel *CLV* pathway members [59]. Plants with the *fasciated and branched* (*fab)* multilocular phenotype contain a missense mutation in the closest tomato homolog of *CLV1*, which affects the kinase domain. Interestingly, the *fasciated inflorescence (fin)* and *fab2* phenotypes are caused by mutations in arabinosyltransferase genes. Arabinosyltransferases catalyze the transfer of l-arabinose to the hydroxyl group of hydroxyproline (Hyp) residues in their target proteins [68]. In *Arabidopsis*, the Hyp^7^ residue of the active CLV3 polypeptide is post-translationally modified with three L-arabinose residues [34,69], and the addition of arabinosylated SlCLV3 peptides can rescue the tomato *fin* phenotype [59]. Therefore, arabinosyltransferase genes are critical components of the CLV-WUS stem cell signaling pathway that can impact crop productivity traits.

## 4. CLV-WUS Pathway in Grasses

The broad function of the CLV-WUS pathway in mediating shoot meristem maintenance is conserved in agronomically important grass species (Table 1, Figure 2C), with some important modifications. In rice *(Oryza sativa* L.), stem cell maintenance appears to be regulated by several distinct pathways, the relative contributions of which depend on the type of meristem. The *FLORAL ORGAN NUMBER (FON1)* and *FON2* genes restrict stem cell accumulation specifically in floral meristems, without affecting vegetative or inflorescence meristem activity [70]. *FON1* encodes the rice ortholog of the CLV1 receptor kinase [62]. It is expressed within the floral meristems but also within the shoot meristem throughout development, suggesting that related receptor kinase genes share functional redundancy with *FON1* in vegetative and inflorescence tissues [62]. Such genes, however, remain to be characterized. The *FON2* gene, also referred to as *FON4*, functions in the same genetic pathway as *FON1* and encodes a CLV3-related protein [58,60]. Like *CLV3*, *FON2* is expressed at the apex of both shoot and floral meristems [58,60]. Thus, in rice floral meristems, the FON1-FON2 system corresponds to the CLV1-CLV3 peptide-receptor kinase signaling system in *Arabidopsis* (Figure 3).

Several other *CLE* genes also play roles in orchestrating rice meristem maintenance (Figure 2C and Figure 3). QTL analysis identified the *FON2 SPARE1 (FOS1)* gene in indica varieties as a suppressor of the *fon2* floral organ number phenotype in japonica, indicating that *FOS1* can substitute for *FON2* activity in rice floral meristems [71]. Constitutive expression of *FOS1* leads to termination of the vegetative SAM, suggesting a potential function for FOS1 in vegetative SAM cell maintenance [71]. The FOS1 CLE domain is more similar to *Arabidopsis* CLE8 and CLE13 than to CLV3, and because FOS1 activity does not require FON1, FOS1 signaling is thought to occur largely in parallel with the FON1-FON2 pathway [71]. Two other *CLE* genes, *FON2-LIKE CLE PROTEIN1 (FCP1)* and *FCP2*, encode proteins that differ in the CLE domain by one amino acid [72] and act redundantly to negatively regulate vegetative stem cell activity and promote leaf initiation [73]. FCP1 represses the expression of rice *WOX4*, an ortholog of *Arabidopsis WOX4* [74], which promotes the undifferentiated state of the vegetative SAM [73]. Thus the rice *WOX4* gene functions similarly to the *Arabidopsis WUS* gene [19], whereas the *WUS* ortholog in rice [74], called *TILLERS ABSENT1 (TAB1)*, is required for axillary meristem initiation but not for shoot or floral meristem maintenance [75]. These studies identify additional *CLE* signaling peptide genes besides *CLV3* as potential targets for genome editing to enhance yield traits in crop plants, particularly grasses.

Maize is a monoecious plant that develops two distinct inflorescence meristem structures: the terminal IFM, called the tassel, that bears male flowers; and the axillary IFMs, called the ears, that bear female flowers. The ear inflorescence meristems produce multiple rows of secondary meristems called spikelet pair meristems, which branch to form spikelet meristems. The spikelet meristems then branch to form two floral meristems, one of which develops into a flower (and after fertilization, a seed kernel) while the other aborts. Modern cultivated corn varieties contain between 8 and 20 rows of kernels within their ears [76], compared to the two rows of kernels found in teosinte, the ancestor of maize, and the ability of the ear IFM to produce additional rows of spikelet meristems appears to have been a major factor in the maize domestication process [5,15]. Molecular evidence indicates that CLV-WUS pathway components underlie much of the variation in this key yield trait.

Mutations at multiple maize loci generate fasciated phenotypes in which the male and/or female inflorescences are enlarged and display increased numbers of spikelet pair and/or spikelet meristems [77]. One of the first such mutants cloned was *thick tassel dwarf1 (td1)*, which displays increased tassel and ear IFM size and results from a mutation in the maize ortholog of the *CLV1* gene [63] (Table 1). The *TD1* locus maps near QTL for tassel spikelet density and for kernel row number [63], whereas the *FASCIATED EAR2 (FEA2)* gene encodes the maize ortholog of *CLV2* [5] and corresponds to a distinct QTL for kernel row number [78]. Thus multiple CLV receptors are likely to have been targets of selection during maize domestication (Figure 2D and Figure 3).

The FEA2 receptor-like protein is proposed to regulate meristem maintenance by transmitting signals from two different CLE peptides through two distinct downstream pathways. FEA2 physically associates in vivo with COMPACT PLANT2 (CT2), the alpha subunit of the heterotrimeric GTP binding protein [79] that along with other Gα domain-containing eXtra Large GTP-binding proteins (XLGs) contribute to restricting IFM size [80]. In CLE peptide response assays both *fea2* and *ct2* plants are resistant to application of ZmCLE7, the maize CLV3 ortholog, suggesting that ZmCLE7 peptide signaling is transmitted across the plasma membrane by a FEA2-CT2 receptor-G protein complex [61]. FEA2 also heterodimerizes with ZmCRN, which acts in separate pathway from CT2. *Zmcrn* plants are sensitive to ZmCLE7 application, but both *fea2* and *Zmcrn* plants are resistant to the application of a related CLE peptide ZmFCP1. In contrast to *ZmCLE7*, *ZmFCP1* is not expressed in the SAM but is detected in incipient and initiating leaf primordia [81]. FEA2, therefore, also appears capable of transmitting a ZmFCP1 signal from organ primordia to regulate IFM activity through a ZmCRN-mediated pathway. Interestingly, the *ZmCRN* locus has significant association with kernel row number variability [82], suggesting that it too contributes to quantitative variation in this trait.

Finally, the CLE peptide ZmFCP1 signals through the LRR receptor-like protein FASCIATED EAR3 (FEA3) to suppress the expression of *ZmWUS1* in the region below the organizing center [81] (Figure 2D). Computational models suggest that ZmFCP1 signaling from developing organ primordia is sufficient to restrict stem cell accumulation in the neighboring SAM by limiting the size of the *ZmWUS1* expression domain [81]. Whether the other maize *WUS* ortholog, *ZmWUS2*, is also a target of ZmFCP1-FEA3 signaling is unknown. FEA3 acts in a separate pathway than FEA2 and weak alleles of *FEA3* and *FEA2* independently enhance kernel row number, although weak *fea2* alleles do not increase overall yield due to a compensatory reduction in kernel size [78,81]. Nonetheless, in maize as in other crop plants, the reduction of stem cell regulatory gene activity can lead to improvement of agronomic traits.

## 5. Perspectives

Gene homologies between *Arabidopsis* and agronomic plants continue to be robust tools for technology transfer, facilitating the translation of basic genetic and genomic information into direct crop improvements. A recent study of the moss *Physcomitrella patens* reveals that the core components of the CLV signaling pathway, namely a CLE peptide and a CLV1/BAM-like RLK, originated with land plants, and that their ability to regulate stem cell proliferation and cell fate is likely to be an ancestral feature of land plants that enabled three-dimensional growth [31]. To date, *CLE* genes have been identified in over 50 plant species, including *Medicago truncatula*, *Lotus japonicas*, wheat, potato, soybean, common bean, banana, and poplar [30]. Additionally, members of the *WUS* clade of *WOX* genes with stem cell-related functions appeared after the divergence of vascular plants from bryophytes [83]. Thus, the potential for modulating the *CLV-WUS* pathway and related *CLE* genes to enhance yield traits exists in a very large number of agricultural plant species.

To date, a major challenge to manipulating yield trait genes in agronomic plants has been the presence of multiple genes within the genome that encode redundant or overlapping stem cell maintenance functions. For example, several homologous copies of the *CLV1*, *CLV2*, and *CLV3* genes exist within polyploid genomes such as *Brassica napa* [50] and wheat *(Triticum aestivum* L.) [84]. In addition, genetic evidence indicates that multiple *CLE* genes as well as multiple *CLV1/BAM* LRR-RLK gene paralogs are involved in the regulation of stem cell maintenance. The advent of multiplex genome editing, which directs the simultaneous targeting of multiple members of a gene family as well as multiple components of a molecular pathway [85], offers great potential to produce beneficial architecture modifications in both dicot and monocot crop species. In this respect, it is worth noting that hypomorphic mutations that reduce *CLV-WUS* gene function, such as mutations in tomato *CLV3* or *WUS* regulatory regions [59,66,67] or missense mutations in maize *CLV1* or *CLV2* receptor kinase genes [78,81], can be sufficient to achieve significant yield increases without the need to completely eliminate gene function. Thus, novel approaches such as genome editing of stem cell maintenance gene promoters [67] may also be a fruitful approach to fine-tune CLV-WUS signaling and thus tailor yield trait optimization within individual crop species.

## Figures and Tables

**Table 1:** *CLV* peptide and receptor gene orthologs in crop plants

Gene Ortholog					
*Arabidopsis*	Brassica	Tomato	Rice	Maize	References
*CLV3*	*BrCLV3*	*SlCLV3*	*FON2*	*ZmCLE7*	[32,54,58,59,60,61]
*CLV1*	*BjMc1; BjLn1*	*FAB*	*FON1*	*TD1*	[36,52,56,62,63]
*CLV2*	*BnA02CLV2; BnC02CLV2*	?	?	*FEA2*	[5,38,50]
*CRN*	?	?	?	*ZmCRN*	[39,61]
